# Do Parents Recognize Autistic Deviant Behavior Long before Diagnosis? Taking into Account Interaction Using Computational Methods

**DOI:** 10.1371/journal.pone.0022393

**Published:** 2011-07-27

**Authors:** Catherine Saint-Georges, Ammar Mahdhaoui, Mohamed Chetouani, Raquel S. Cassel, Marie-Christine Laznik, Fabio Apicella, Pietro Muratori, Sandra Maestro, Filippo Muratori, David Cohen

**Affiliations:** 1 Department of Child and Adolescent Psychiatry, AP-HP, Groupe Hospitalier Pitié-Salpêtrière, Université Pierre et Marie Curie, Paris, France; 2 Institut des Systèmes Intelligents et de Robotique, CNRS UMR 7222, Université Pierre et Marie Curie, Paris, France; 3 Department of Child and Adolescent Psychiatry, Association Santé Mentale du 13ème, Paris, France; 4 Division of Child Neurology and Psychiatry, Stella Maris Scientific Institute, University of Pisa, Calombrone, Italy; The University of Queensland, Australia

## Abstract

**Background:**

To assess whether taking into account interaction synchrony would help to better differentiate autism (AD) from intellectual disability (ID) and typical development (TD) in family home movies of infants aged less than 18 months, we used computational methods.

**Methodology and Principal Findings:**

First, we analyzed interactive sequences extracted from home movies of children with AD (N = 15), ID (N = 12), or TD (N = 15) through the Infant and Caregiver Behavior Scale (ICBS). Second, discrete behaviors between baby (BB) and Care Giver (CG) co-occurring in less than 3 seconds were selected as single interactive patterns (or dyadic events) for analysis of the two directions of interaction (CG→BB and BB→CG) by group and semester. To do so, we used a Markov assumption, a Generalized Linear Mixed Model, and non negative matrix factorization. Compared to TD children, BBs with AD exhibit a growing deviant development of interactive patterns whereas those with ID rather show an initial delay of development. Parents of AD and ID do not differ very much from parents of TD when responding to their child. However, when initiating interaction, parents use more touching and regulation up behaviors as early as the first semester.

**Conclusion:**

When studying interactive patterns, deviant autistic behaviors appear before 18 months. Parents seem to feel the lack of interactive initiative and responsiveness of their babies and try to increasingly supply soliciting behaviors. Thus we stress that credence should be given to parents' intuition as they recognize, long before diagnosis, the pathological process through the interactive pattern with their child.

## Introduction

### Early signs of autism

Autism is a severe psychiatric syndrome characterized by the presence of abnormalities in reciprocal social interactions, abnormal patterns of communication, and restricted and stereotyped behaviours starting before age 3 [1]. Autism is now a well-defined clinical syndrome after the third year of life, and considerable progress in understanding its emergence in the first two years of life has been achieved [2], [3]. Although there have been significant advances in describing single or multiple early signs, our ability to detect autism during early age is still challenging. Home movies (ie., naturalistic films recorded by parents during the first years of life) and direct observations of at risk infants are the two most important sources of information for overcoming this problem. They have both described children with autism disorder (AD) during the first 18 months as not displaying the rigid patterns described in older children. In particular, AD children can gaze at people, turn toward voices and express interest in communication as typically developing (TD) infants do [4], [5]. However, in several studies, children who later develop AD show as early as the first year less social behavior (e.g., looking at others, especially at the face), communication skills (e.g., responding to name), inter-subjective initiative, and emotion expression than TD infants. In the second year, early social signs intensify; expressive and receptive language fails to develop, while the lack of inter-subjective skills and of emotional expression persists [4], [5]. These insights from home movies have been confirmed in studies of at risk children [6], [7], [8], [9] and in studies using retrospective data from parental interviews to assess early signs of AD (Guinchat et al., in revision). As regards specificity, signs that differentiate AD children from children with intellectual disability (ID) are limited to the second year: fewer responses to name, fewer glances to others, lower eye contact quality and quantity, less positive facial expression and fewer inter-subjective behaviors (e.g., showing shared attention) [4], [5]. To further investigate early signs in the interactive field, Muratori et al. [10] studied home movies of the first three semesters of life from AD, ID and TD children with independent scoring of both baby (BB) and caregiver (CG) behaviors and timing. AD infants displayed impairments in “syntony”, “maintaining social engagement”, “accepting invitation” and in “orienting to their name” (definitions are given in Table 1) as early as the first year of life in comparison with TD children. At semester 3, some items differentiated AD from TD while for other items AD showed significantly lower scores compared to ID. In addition, they noted that AD babies received less action than ID from their CG to regulate down their arousal and mood.

**Table 1:** Infant's and caregiver's behaviors and meta-behaviors from the infant caregiver behavior scale (ICSB).

Meta-behavior	Item Behavior	Glossary
***Child Behaviors (N = 29)***		
Behavior with object	Orienting toward object	The child directs his/her gaze towards a source of new sensory stimulation coming from an object
	Gaze Following an object	The child shifts his/her gaze to follow the trajectory of an object.
	Explorative activity with object	The child touches something by hands, mouth or other sensory-motor actions, to find out what it feels like.
	Looking at object/around	The child directs his/her eyes towards an object, or simply looks around.
	Smiling at object	The child intentionally smiles at object.
	Enjoying with object	The child finds pleasure and satisfaction experiencing a physical or visual contact with an object.
	Seeking contact with object	The child employs spontaneous and intentional movements to reach contact with an object.
Vocali-zations	Simple Vocalisation	The child produces sounds towards people or objects.
	Crying	The child starts crying after a specific/non specific event.
Orienting toward people	Orienting toward people	The child directs his/her gaze towards a source of new sensory stimulation coming from a people
	Gaze Following a person	The child shifts his/her gaze to follow the trajectory of another person.
	Explorative activity with person	The child touches a person to find out what it feels like (by hands, mouth or other sensory-motor actions).
Receptive to people	Looking at people	The child directs his/her eyes towards a human face.
	Smiling at people	The child intentionally smiles at a person.
	Enjoying with person	The child finds pleasure and satisfaction experiencing a physical or visual contact with a person.
	Sintony *	The child shows signs of congruous expressions to affective solicitations, to the other's mood.
Seeking people	Seeking contact with person	The child employs spontaneous and intentional movements to reach contact with a person.
	Soliciting	The child displays a vocal or tactile action to attract the partner's attention or to elicit another response.
Inter-subjective behavior	Anticipation of other's intention	The child makes anticipatory movements predicting the other's action.
	Communicative gestures	The child displays use of social gestures.
	Referential gaze	The child shifts his/her gaze towards the caregiver to look for consultation in a specific situation.
	Gaze following gaze	The child shifts his/her gaze to follow the gaze of another person.
	Accept Invitation	The child's behavior is attuned to the person's solicitation within 3 seconds.
	Orienting to name prompt	The child assumes a gaze direction towards the person who calls him/her by the name.
	Imitation	The child repeats, after a short delay, another person's action.
	Pointing comprehensive/ declarative/requestive	The child a) shifts his/her gaze towards the direction pointed by a person; b) points something in order to share an experience; c) in order to obtain an object.
	Maintaining social engagement *	The child takes up an active role within a two-way interaction in order to keep the other person involved. The child interacts, vocalises and maintains turn taking.
	Meaningful Vocalisation	The child intentionally produces sounds with a stable semantic meaning
***Caregiver's Behaviors (N = 8)***		
Reg-up/down	Regulation up * /down	Modulates the child's arousal and mood, to either excite (reg-up) or calm (reg-down).
Touching	Touching	Stimulates the child requesting attention by touching him/her.
Vocalization	Vocalizing/naming/behavior request	Stimulates the child requesting attention by vocalizing, naming
Gesturing-showing	Gesturing/showing object	Stimulates the child requesting attention by gesturing or showing him object

### Taking into account interaction

One of the main limitations of these studies is that they have not or only poorly taken into account the importance of BB/CG synchrony and reciprocity in the early interactions [11]. As it is of seminal importance to have more insight not only into early social competencies of infants who are developing autism but also into interactive situations where they preferentially emerge, we tried to overcome these caveats by using for previous data [10] new engineering techniques of interaction analysis focusing on reciprocity and synchrony between BB and CG. Recently, applying machine learning methods to explore TD infant and mother behavior during interaction, Messinger et al. [12] showed that developmental changes were most evident when the probability of specific behaviors was examined in specific interactive contexts. The aims of the current study were to assess early social interactions of infants with TD, ID and AD taking into account simultaneously: CG behavior, BB behavior, synchrony of the interaction partners, and finally, the two directions of interaction (from CG to BB and from BB to CG). Among others, we hypothesized that (1) infants with AD should exhibit a growing deviant social development whereas those with ID should rather show an initial delay of development; (2) CG of babies with atypical development should feel very early the initial pathological process and this feeling could be expressed through atypical/unusual interactive patterns.

## Materials and Methods

### General view of the study

**Figure 1:**
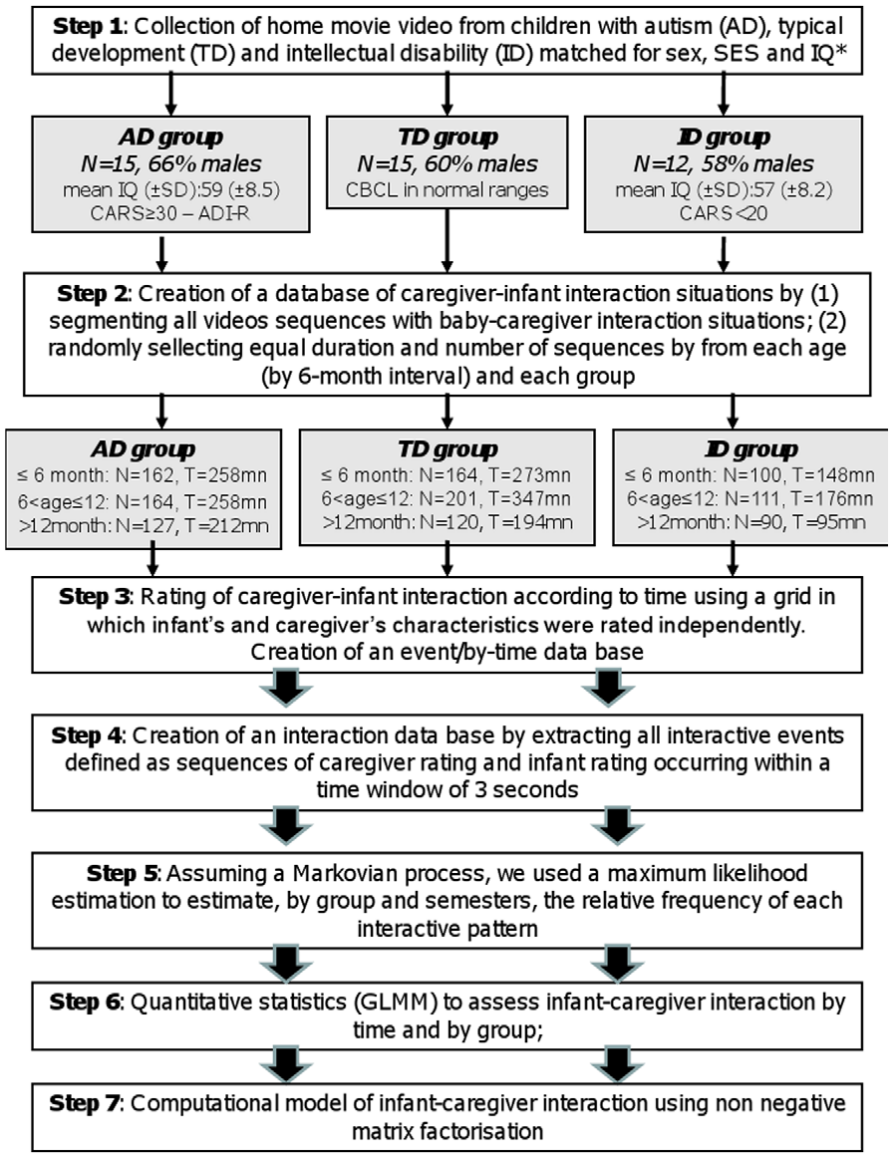
Diagram flow of the study. SES = Socio Economic Status; IQ = Intellectual quotient; CARS = Children Autism Rating Scale; CBCL = Child Behavior Check List; SD = Standard Deviation; GLMM = Generalized Linear Mixed Model; *IQ matching only between ID and AD children and based on Griffiths Mental Developmental Scale or Wechsler Intelligent Scale.

The diagram-flow of the study is summarized in Figure 1. Forty-two children were randomly selected inside the Pisa Home Movie database, with the following criteria: 15 who will be diagnosed with AD, 12 with ID and 15 who will develop normally (step 1). All scenes showing a situation in which social interaction could occur (i.e. all scenes with an infant and an adult) were extracted and, if necessary, segmented in short sequences in order to be scored (step 2). CG and BB behaviors were rated independently within each interaction sequence according to a grid with a specific part for each partner (step 3). An interaction database was created by extracting [CG→BB] or [BB→CG] signals occurring “simultaneously”, that is within a time window of 3 seconds (step 4). A computational model using Markov assumption of interaction was performed to describe the interaction (step 5). Quantitative statistics were performed to assess and compare emergence of interactive patterns by time and by group (step 6). To study these interactive patterns with an integrative perspective, Non-negative Matrix Factorization (NMF) were performed (step 7). Steps 1, 2, and 3 have been described in a previous report where a full description is available [10]. Here we only summarize them.

### Participants (Step 1)

The study has been approved by the Ethical Committee of the Stella Maris Institute/University of Pisa, Italy [13]. The Pisa Home Movie data base includes three groups of children matched for gender and socio-economic status, with home movies (HM) running for a minimum of 10 minutes for each of the first 3 semesters of life. Group 1 includes 15 children (M/F: 10/5) with a diagnosis of AD without any sign of regression confirmed with the Autism Diagnostic Interview Revised [13]. Group 2 includes 12 children (M/F: 7/5) diagnosed with ID according to the DSM-IV criteria and a Childhood Autism Rating Scale (CARS) [14] total score under 25. The composite IQ score was below 70 for both AD and MR (figure 1). Group 3 includes 15 children (M/F: 9/6) with a history of typical development confirmed by non pathological scores at the Child Behavior Check List [15].

### Extraction of CG-BB interaction situations (Step 2)

An editor, blind to children diagnoses, selected from among the HM of each child all segments running for at least 40” where the infant was visible and could be involved in human interaction (standard situations). For each infant, the sequences were organized in three periods of 6 months of age (≤6 month; 6<age≤12 months; >12 months). Sequences were randomly selected by group and by semester. Preliminary t-test analysis showed that chosen video material was comparable across groups and for each range of age, in length and number of standard situations.

### Computer-based coding system (Step 3)

The Observer 4.0® was configured for the application of the Infant Caregiver Behavior Scale (ICBS) to the video media file-material. The ICBS (Table 1) is composed of 29 items referring to the ability of the BB to engage in interactions and 8 items describing CG solicitation or stimulation toward the infant to obtain his attention. All target behaviors were described as Events which take an instant of time. Caregiver regulation up caregiver regulation down were described as events and also states which take a period of time and have a distinct start and an end.

Four coders were trained to use the computer-based coding system until they achieve a satisfactory agreement (Cohen's Kappa ≥0.7). The standard situations derived from the HM of the three groups of children (AD, ID and TD) were mixed, and each one was rated by one trained coder blind to which group they belonged. For a continuous verification of inter-rater agreement, 25% of standard situations were randomized and rated by two coders independently. The final inter-rater reliability, calculated directly by the Observer, showed a satisfactory Cohen-κ mean value ranging from 0.75 to 0.77.

### Creation of the interaction database (step 4)

We first created an interaction data base (Step 4) by extracting all interactive events defined as sequences of caregiver behavior and infant behavior co-occurring within a time window of 3 seconds. The whole interaction database was divided into two sets: (1) CG→BB interactions, i.e. any child behaviors occurring within the 3 seconds following any caregiver behavior (including events that occur within the same second); (2) BBCG interactions, i.e. any caregiver behaviors occurring within the 3 seconds following any child behavior (again including concomitant events). The 3 second window was based on available literature on synchrony ([11]). Interactive events that occurred at the same second were integrated in the two sets of the interaction database because it was too difficult to assume who was primary or secondary in the interaction. Extraction was performed using Linux based script. The sequence of n interactive patterns is termed n-gram as usually done in natural language processing or gene analysis. In this study, we only focused on bi-gram modeling. Given the large number of possible types of interaction ([CG item x BB item] combinations  =  8×29), and the low frequency of several items in the data base, we created five CG meta-behaviors (Vocal solicitation, Touching, Gestural solicitation, Regulation up, Regulation down) and six BB meta-behaviors (Vocalizations, Inter-subjective behavior, Seeking people, Receptive to people, Orienting toward people, Behavior with object) by grouping ICBS items. Meta behaviors are shown in the left column of Table 1. Then we repeated the process of extraction to obtain finally, for each standard situation, all sequences of caregiver meta-behavior and infant meta-behavior occurring within a time window of 3 seconds.

### Characterization of infant-caregiver interactive patterns (Step 5)

**Figure 2:**
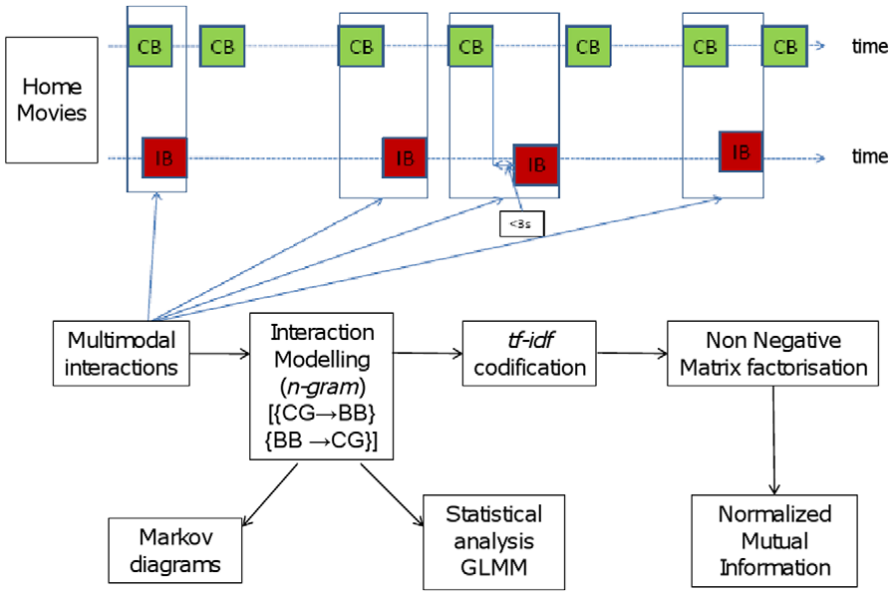
Analysis of parent-infant interaction: general principals. {CG→BB} ensemble of interactive patterns from caregiver (CG) to baby (BB); {BB→CG} ensemble of interactive patterns from baby (BB) to caregiver (CG); GLMM = Generalized Linear Mixed Model.

General principles of the analysis we used to investigate interactive patterns by group and by time are summarized in figure 2. First, we aimed to describe infant-caregiver interaction by time and by group and assess emergence of language and social engagement by time and by group as they are core issues of autism. For each of the two sets of the database (ie., the two directions of interaction), assuming a Markovian process, we used a maximum likelihood estimation to estimate, by group and semesters, the probability (relative frequency) of each interactive pattern or bi-gram (couple of CG and BB items) using meta behaviors only (6×5 for BB→CG and 5×6 for CG→BB). Grouping all the more frequent (>1%) interactive patterns (or bi-grams) allows designing Markov chains representing the parent-infant interaction. Markov diagrams were performed using Graphviz (see http://www.graphviz.org/).

### Quantitative statistics (Step 6)

Statistical analyses were performed using R Software, Version 2.7 (The R Foundation for Statistical Computing). Analyses were conducted separately on each of the two sets of the data base (CG→BB and BB→CG). We computed descriptive statistics of each CG and BB interactive behavior and meta-behavior, by group and by semester. To assess by group and/or by time significant associations, we used a generalized linear mixed model (GLMM). Using this model, we performed a linear regression that was generalized to the variable distribution (here a quasi Poisson distribution) and with a random effect to take into account patients' auto correlations [16]. The distribution of each item behaviors and meta-behaviors was studied in order to compute statistics with GLMM. All BB and CG *meta behaviors*, 6 CG *items* (Gesturing, Showing object, Vocalizing, Request Behavior, Naming) and 9 BB *items* (Orienting to name, Exploring object, Looking at object, Looking around, Looking at People, Contact Object, Orienting to People, Simple Vocalizations, Smiling at People) satisfied a “quasi-Poisson” law. Several other items occurring with a low frequency were not statistically usable because their distribution did not satisfy any known law. All BB and CG items and meta behavior responding to a quasi Poisson distribution were included in the model.

We conducted two univariate analyses first with Group as independent variable for a given semester, and then Time (semester) as independent variable within the same group. Then a multivariate analysis with both Time and Group was performed. As we knew that (1) AD and ID children would not behave better in interaction than TD and that (2) interactive behaviors change with time in pathological and typical children, we used a one-tail threshold of significance (t = 1.645 for p = 0.05) for each calculation of p.

### Computational model of infant-caregiver interaction (Step 7)

Modeling and analyses done by Markov chains and GLMM provide useful insights on dynamic and relevance of individual interactive patterns. In order to study these interactive patterns with an integrative perspective, we proposed to employ a more global approach using Non-negative Matrix Factorization (NMF) [17]. All the *m* interactive patterns among the *n* movies have been grouped into a matrix V.

NMF is an unsupervised feature extraction method involving the decomposition of a non-negative matrix V (dimension *n* x *m*) into two non-negative matrices W (*n* x *k*) and H (*k* x *m*) by multiplicative updates algorithm:
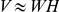



The non-negativity constraints are relevant for the analysis of human behaviors since they allow only additive, not subtractive, combinations (part-based representation). The rank *k* of the factorization represents the number of latent factors and is usually chosen such that *(n+m)k<nm*. The rank *k* is interpreted as the number of clusters resulting in groups of interactive behaviors. Indeed, rows or columns of the decomposed matrices (H and W) are usually considered to be the membership degree to a cluster. NMF has been successfully used in various applications including interpretation of social behaviors [18] and computational biology [19]. Most of the studies have pointed important requirements such as the pre-processing of the data, optimization of the rank of factorization (the number of clusters) and also the initialization.

Regarding the pre-processing, we used a method usually employed in document analysis: tf-idf (term frequency-inverse document frequency) [20]. This approach is based on the fact that a query term that occurs in many documents may not be discriminant and consequently should be given less weight than one that occurs in few documents. In our work, terms refer to interactive patterns while documents refer to home movies. The key idea is to give more importance to an interactive pattern in a given home movie if 1) the interactive behavior appears frequently in the home movie and 2) the interactive behavior does not appear frequently in other home movies. For a given interactive behavior *t_i_* within a movie *d_j_*, we estimated the term-frequency *tf_ij_*:
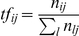
where n_ij_ is the number of occurrences of the considered interactive pattern (t_i_) in the movie d_j_, and the denominator refers to the total of occurrences of all the interactive patterns in the movie d_j_.

The inverse document frequency is a measure of the general importance of the interactive pattern (a measure of informativeness) defined as the logarithm of the ratio of documents (movies) to the number of documents containing a given term (interactive patterns):
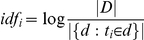
where |D| is the total number of movies in the database and |{d:ti ∈ d }| is the number of movies containing the interaction pattern ti. Finally, the tf-idf representation is obtained by multiplying the weights: (tf-idf)ij  =  tfij x idfi.

The number of clusters is an important issue in the current work since it will provide insights on the combination of interactive patterns among groups and semesters. To determine the optimal k which decomposes the samples into ‘meaningful’ clusters, we investigated ‘Homogeneity-Separation’ since the standard definition of a good clustering is that of ‘Homogeneity-Separation’: every element in a cluster must be highly similar (homogeneous) to the other elements in the same cluster and highly dissimilar (separation) to elements outside its own cluster.

The stochastic nature of NMF requires strategies to obtain stable and reliable results that also depend on the initialization process. In the current work we use a recent method proposed by Boutsidis and Gallopoulos [21] termed Nonnegative Double Singular Value Decomposition (NDSVD), which is based on Singular Value Decomposition (SVD) but with non-negative constraints. Unlike random approaches, NDSVD guaranties stable results but not necessarily efficient ones; for this purpose multiple runs of NDSVD have been carried out.

In order to understand the developmental similarity of AD children towards TD, and ID children towards TD, we calculated the value of the Normalized Mutual Information (NMI) as proposed by Strehl and Ghosh [22]. The NMI of two different clustering measures the agreement between the two clustering:
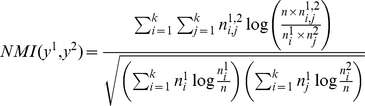
where 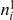 is the number of interactive patterns belonging to cluster c_i_ using clustering y^1^, 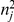 is the number of interactive patterns belonging to cluster c_j_, using clustering y^2^, and 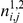 is the number of interactive patterns belonging to cluster c_i_, using clustering y^1^ and belonging to the cluster c_j_ using y^2^. One should note that NMI(y^1^,y^1^) = 1 indicating same clustering and consequently same interactive behaviors.

## Results

### Early interaction in TD children and significant developmental changes

**Figure 3:**
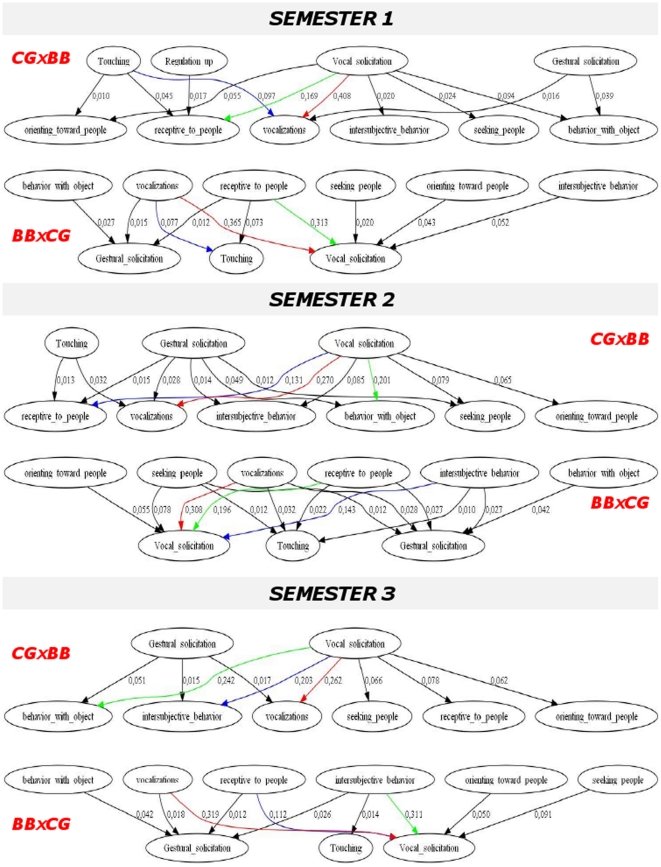
Markov diagram of the main early interactive patterns in typical developing children according to time and interaction direction.

Figure 3 summarizes the Markov diagram of all interactive patterns in TD children (at the meta-behavior level) occurring with a frequency higher than 1% according to both interaction direction [CG→BB] or [BB→CG] and semester. The diagram estimates 93.6% to 96% of the total interaction patterns according to semester and direction of interaction. When CG starts interaction, he/she predominantly uses vocal solicitation at all semesters. BB responds with vocalization (38.6%), being receptive to people (16%) and with object behaviors (8.9%) during the first semester (S1). BB responds with vocalization (25.4%), with object behaviors (18.8%) and being receptive to people (12.4%) during S2. BB responds with vocalization (24.6%), with object behaviors (22.9%) and intersubjective behaviors (19.1%) during S3. When BB starts interaction he uses preferentially vocalizations and being receptive to people during S1, to which CG answers with vocalizations (54.8%) and touching (12.1%). During S2, BB uses behavior with object (28.8%), vocalizations (26.9%), being receptive to people (17.8%) and intersubjective behaviors (12.4%). CG answers predominantly with vocal solicitation. During S3, patterns are similar but BB intersubjective behaviors (21.9%) are much more frequent than being receptive to people (7.3%).

**Figure 4:**
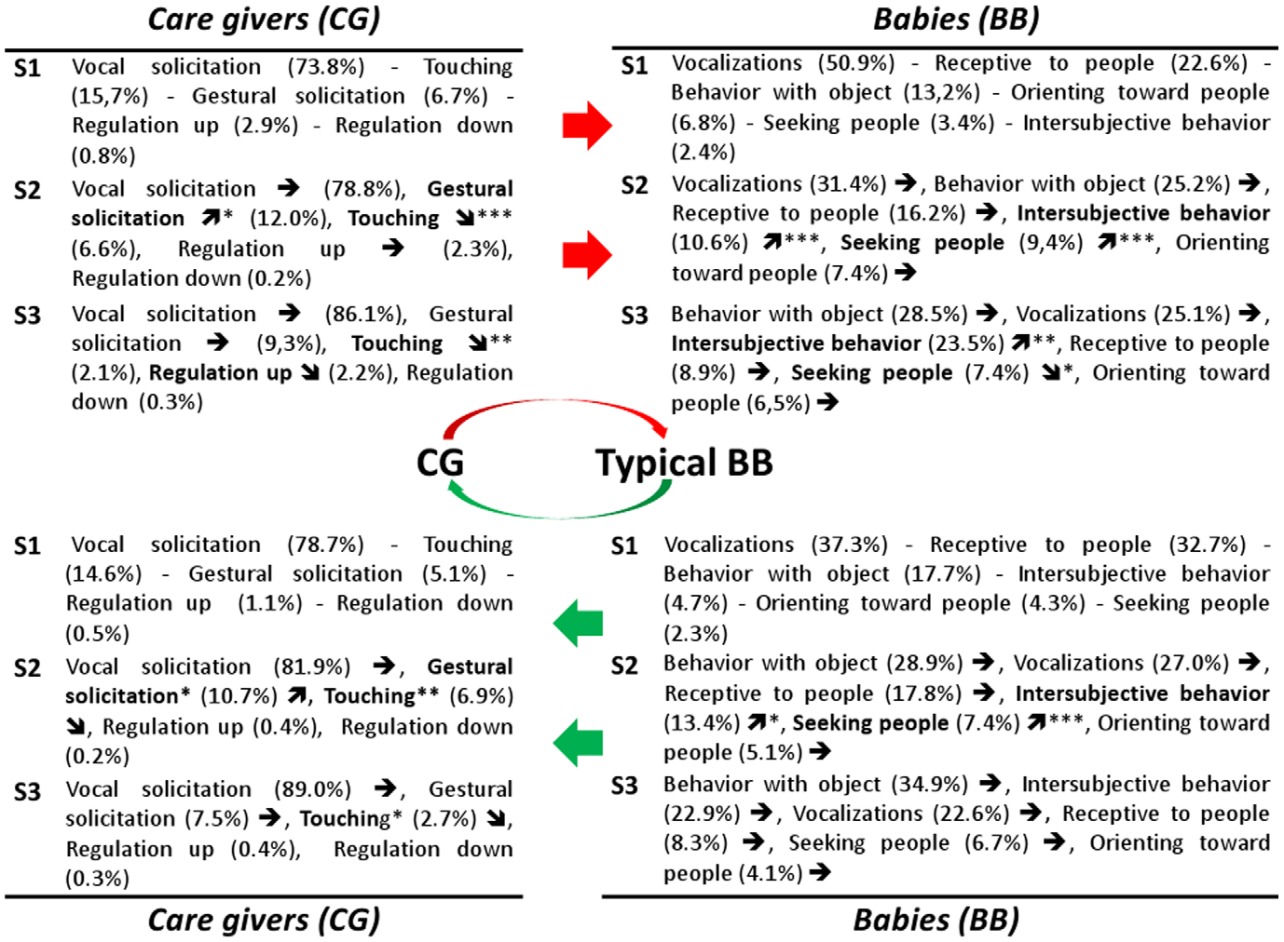
Developmental view of meta-behaviors for typical infants. Top: Care-Givers towards Babies/Down: Babies towards Care-Givers. S =  Semester; See Table 1 for a brief description of cited infant's or care-giver's behaviors and meta-behaviors. In brackets: % of this behavior inside the whole interactions of the group in the semester. The arrow indicates behaviors that significantly grow (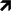) or decrease (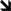) compared with the previous semester (*p<0.05; **p<0.01; ***p<0.001).

For each interaction direction, figure 4 shows the relative distribution of meta-behaviors by semester, and summarizes the GLMM model in TD children. Significant developmental changes are indicated by an arrow (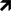 or 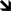 according to a significant increase or decrease). They are as follows: BB intersubjective behaviors and seeking people behaviors, both as interaction initiation [BB→CG] and response [CG→BB] increase from S1 to S2. The increase continues from S2 to S3 as response [CG→BB] for BB intersubjective meta-behavior whereas BB seeking people behaviors decrease (only as response, too). However, during S3, BB intersubjective behaviors become the second child solicitation for CG. BB behavior with object becomes the first solicitation from the BB as soon as S2, and also the first response of the BB at S3. CG touching behaviors decrease in both directions from S1 to S2, and from S2 to S3. CG gestural solicitation increases from S1 to S2. CG vocal solicitation is predominant in all semesters. CG regulation up/down are very low in TD children during interactive patterns.

For the meta-behaviors that showed significant changes during early development, we also tested the corresponding CGBB individual items included in the model (see methods). Significant results are as follows: BB orienting to name increases (p<0.001) from S1 to S2 and decreases from S2 to S3 (p<0.001); BB contact object increases (p<0.05) from S1 to S2; BB exploring object increases (p<0.001) from S1 to S2 and again from S2 to S3 (p<0.001); BB looking around (p<0.05) and BB smiling at people (p<0.05) decrease from S2 to S3. CG gesturing increases (p<0.001) from S1 to S2 and then decreases (p<0.001) from S2 to S3; CG request behavior (p<0.05) and CG naming (p<0.01) increase from S1 to S2.

### Early interaction in AD and ID infants compared to that in TD infants

**Figure 5:**
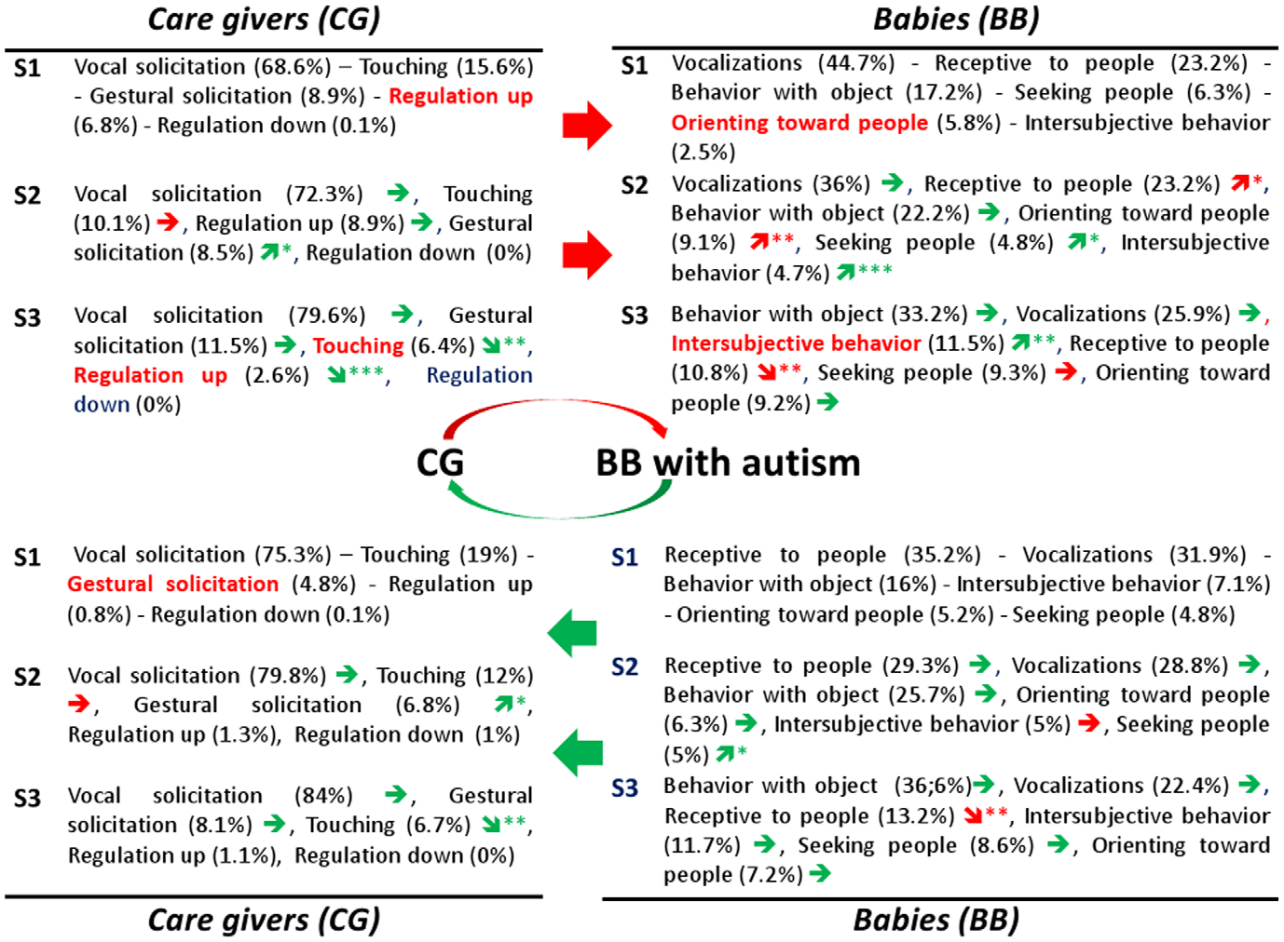
Developmental view of main interactive behaviors for infants with autism. Top: Care-Givers towards Babies/Down: Babies towards Care-Givers. S =  Semester; See Table 1 for a brief description of cited infant's or care-giver's behaviors and meta-behaviors. In brackets: % of this behavior inside the whole interactions of the group in the semester. The arrow indicates behaviors that significantly grow (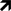) or decrease (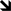) compared with the previous semester (*p<0.05; **p<0.01; ***p<0.001). The red color indicates a significant difference when compared with TD: behavior in red color means that it differs in a group comparison (inside a given semester); arrow in red color means that the progression over time differs from that of the TD children (meaning the arrow has not the same direction). Significant p values are given in the text.

**Figure 6:**
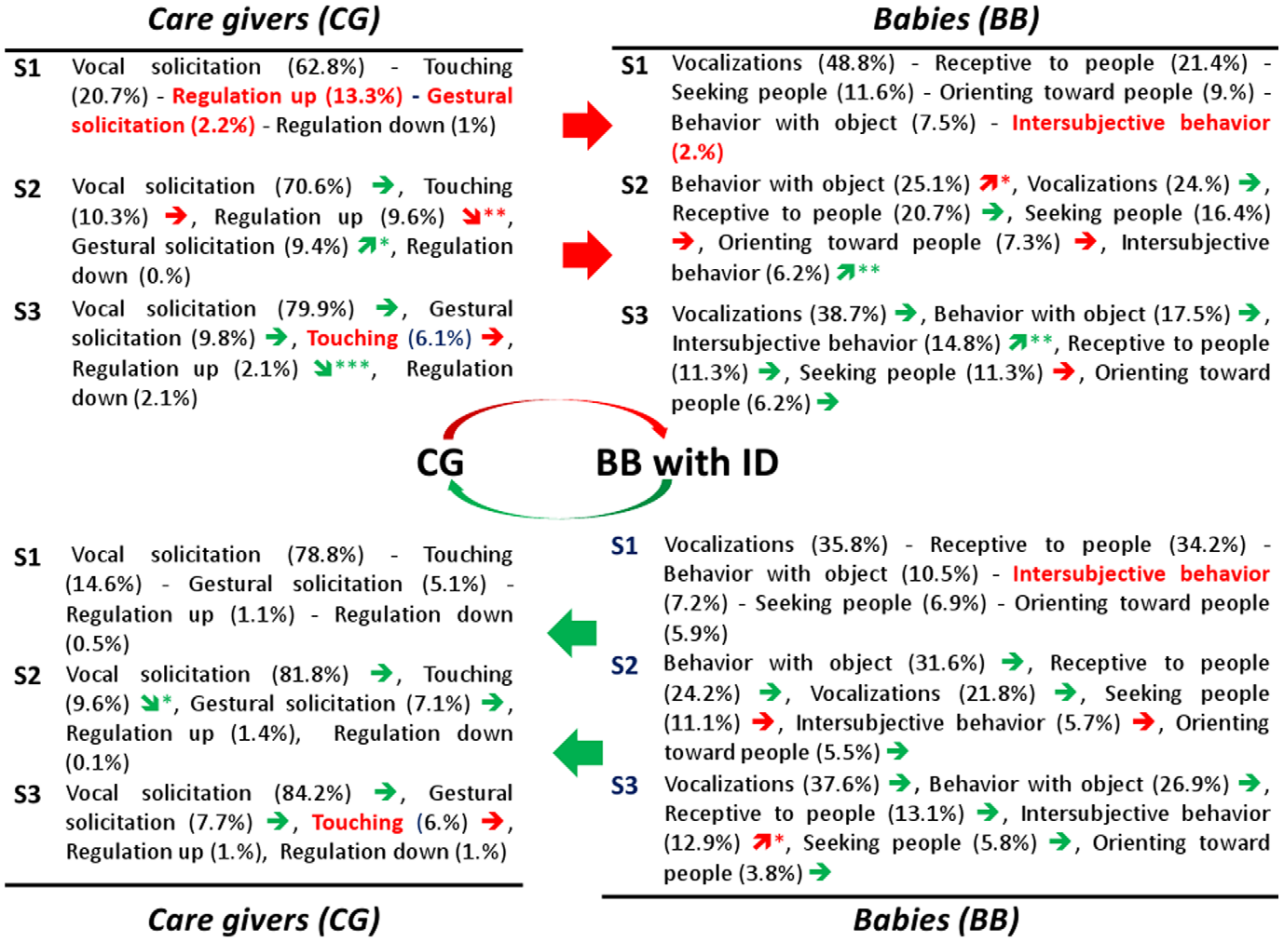
Developmental view of main interactive behaviors for infants with intellectual disability (ID). Top: Care-Givers towards Babies/Down: Babies towards Care-Givers. S = Semester; See Table 1 for a brief description of cited infant's or care-giver's behaviors and meta-behaviors. In brackets: % of this behavior inside the whole interactions of the group in the semester. The arrow indicates behaviors that significantly grow (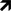) or decrease (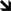) compared with the previous semester (*p<0.05; **p<0.01; ***p<0.001). The red color indicates a significant difference when compared with TD: behavior in red color means that it differs in a group comparison (inside a given semester); arrow in red color means that the progression over time differs from that of the TD children (meaning the arrow has not the same direction). Significant p values of group comparisons are given in the text.

Figure 5 and figure 6 summarize the significant developmental changes over time (represented by an arrow) and the significant differences in the multivariate analysis (by group and by time comparison) using the GLMM model in AD and ID children, respectively.

Considering first child behavior, when CG starts interaction [CG→BB], BB inter-subjective behaviors grow every semester (p<0.01) whatever the group, but they are lower for ID than TD (p<0.01) at S1. In contrast, for AD it is lower (p<0.05) globally (all semesters combined) and tend to be significantly lower (p<0.1) at S3. When BB starts interaction [BBCG], BB inter-subjective behavior is again significantly lower (p<0.05) for ID than TD at S1. From S1 to S2, unlike for TD, BB inter-subjective behavior does not increase in both pathological groups, but only children with ID exhibit a significant increase of inter-subjective behavior from S2 to S3. BB orienting toward people is lower (p<0.05) in response at S1 for AD than TD. However, it significantly increases (p<0.01) from S1 to S2 for AD (whereas TD keep stable). Other BB meta-behaviors (vocalizations, seeking people, being receptive to people, behavior with object) show no significant differences between groups.

From a developmental point of view, AD children, unlike TD children, show a significant increase (p<0.05) of receptive behaviors from S1 to S2, and conversely, a much smaller increase of seeking people behaviors (p<0.05) than TD (p<0.001). In summary, from S1 to S2, AD children become more “open” (receptive) and interested in an exchange (orienting toward people) but only in a passive way (not seeking people); moreover at S3, the decrease of BB receptive behaviors is striking in AD (p<0.01) whereas this is not significant for TD children.

ID children do not show any increase of BB seeking people over time but have high rates at S1. Like TD children but unlike AD children, ID children don't exhibit significant changes over time either in BB receptive behaviors or in BB orienting toward people. Unlike AD and TD children, ID children exhibit a significant increase of BB behaviors with object from S1 to S2, but whatever the semester they stay (but not significantly) below TD and AD.

Considering now CG behavior, CG vocal solicitation is always higher for parents of TD children, but it never reaches significance between groups nor over time. CG gestural solicitation is lower at S1 in the two pathological groups reaching significance for parents of ID children only in initiation [CG→BB] (p<0.05) and for parents of AD children only in response [BB→CG] (p = 0.01). However, for the three groups it increases significantly from S1 to S2 in both ways of interaction, except in response for parents of ID children. CG touching behavior does not change in CG of AD and ID children from S1 to S2, while it decreases for parents of TD children (p<0.001). Then from S2 to S3, it decreases in parents of AD children as it does for parents of TD children. However at S3, CG touching is higher for parents of AD and ID children compared with TD children, in initiation [CG→BB] (p<0.05) and with a tendency (p<0.05 for ID and p<0.1 for AD) in response [BB→CG]. Finally, CG regulation-up duration is higher for parents of ID and AD children (p<0.05) at S1. Then it decreases (p<0.05) from S2 to S3 in all groups. However, at S3, it remains higher (p<0.05) for parents of AD children.

For item behaviors included in the model (see methods), all semesters together (in the multivariate analysis), BB orienting to name and BB exploring object appear lower in the AD group than in TD (p<0.01 and p<0.001 respectively). With regards to the ID group, BB looking object, BB looking around and CG gesturing appear lower than in the TD group (p<0.05). BB exploring object, at S2 and S3, was lower for AD children (p<0.05 and p<0.01 respectively). As for other developmental changes for AD children, from S1 to S2, unlike for TD, BB orienting toward people and BB smiling to people are growing (p<0.01 and p<0.05 respectively). From S2 to S3, unlike for TD children, BB exploring object and BB looking around don't increase, and BB looking at people decreases (p<0.05). From S1 to S2, CG touching increases non-significantly (while there is a significant decrease in TD group: p<0.001) and from S2 to S3, CG gesturing doesn't decrease, and CG naming decreases (p<0.05). For other items, AD group follows a development similar to that of typical.

### Developmental similarity between AD vs TD and ID vs TD using Non negative Matrix Factorization

To give a more general view of interactive patterns during infancy, we also used non- negative matrix factorization. First, we applied a tf-idf (term frequency-inverse document frequency) to transform the scenes annotations into a representation suitable for the clustering task. The best solutions of behavior signals clustering for the ‘Homogeneity-Separation’ method yielded the following number of clusters according to semester (S1, S2, S3): 11, 14 and 9 for TD; 5, 11, 14 for ID; 12, 8, 10 for AD.

**Figure 7:**
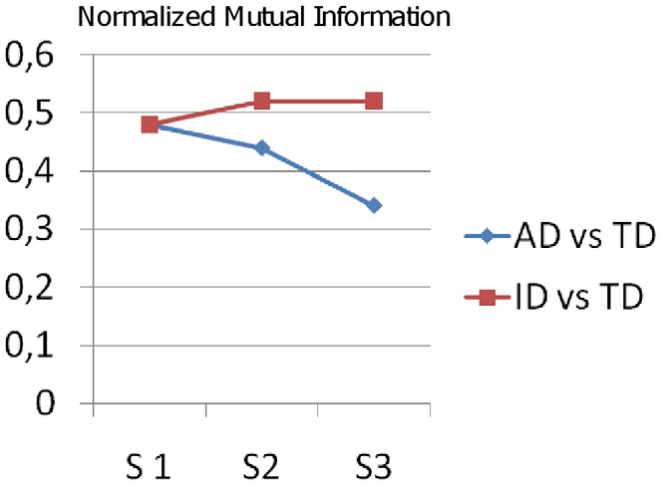
Developmental similarity between intellectual disability (ID) and typical development (TD) (red line) and between autism disorder (AD) and typical development (blue line) using Normalized Mutual Information (NMI) after non negative matrix factorization (S = semester).

To illustrate the developmental similarity of AD children towards TD, and ID children towards TD, we calculated Normalized Mutual Information (NMI) values between the clustering results of TD/AD at each semester (0.48, 0.44, 0.37 for S1, S2, S3 respectively) and NMI values between the clustering results of TD/ID at each semester (0.48, 0.50, 0.47 for S1, S2, S3 respectively). Figure 7 shows that NMI values between the clustering results of TD/AD decrease over time, whereas NMI values between the clustering results of TD/ID show stability over time (see figure 7).

## Discussion

As opposed to all previous home movies studies, the use of engineering methods related to social signal processing allowed focusing on dynamic parent↔infant interaction instead of single behaviors of the baby or of the parent. The focus on interaction has many advantages. First, it allows to maintain attention on antecedents and consequences of interactive behaviors; second it allows to point out significant sequences that could be able to prompt or inhibit social interaction in a naturalistic and spontaneous way; third, it could produce insights for treatments based on parent-infant engagement that are now considered to be a fundamental part of many types of treatment. We discuss our results separately with regard to typical and atypical developments of interactive patterns. Throughout the discussion we put a series of comparisons with results described in a previous paper on the same subjects with the objective to demonstrate the added value of a research on autism using engineering methods which has its focus on interactive social sequences and not just on simple, or even complex, behaviors.

### Summarizing CG↔BB interactive patterns in typically developing babies

Among BB behaviors vocalizations are predominant from birth, and exploring object grows significantly every semester until behaviors with object become the first BB meta-behavior in the second year. While seeking people peaks significantly at second semester compared with next and previous semesters, inter-subjective behavior continues to grow significantly over the semesters. Thus in the second semester, a typical child is rather seeking and attending to his care-giver and little by little turns to objects, even inside the interaction (since our “filter” keeps only behaviors that are included in an interactive dynamic). This pattern describes the typical development of shared or joint attention [23], [24] and points out how this phenomenon is entangled with both the simultaneous increase of inter-subjectivity and with vocalizations.

Also among CG behaviors, vocalizations are predominant from birth. We can assume that this type of stimulation which has its roots in animal communication is the more powerful way to strengthen child attention and affective communication. Probably it happens thanks to prosodic cues specific of infant directed speech [25], [26] that are the object of a parallel paper where we have proposed a specific technological analysis of motherese [27]. Moreover, vocalizations pose the basics of language acquisition along with gestures [28], [29]. Indeed, CG gestural solicitations increase during the first year. In contrast, touching decreases every semester so that as the child becomes gradually more active (seeking people) and conscious (intersubjective acts) in the relationship, parents follow suit by leaving their touching behavior but not their vocalizations and increasing their gestural communication [30]. Indeed, the literature shows that mothers tailored their communication to infants' level of lexical-mapping development [28].

### What differs in AD and ID developments of interactive patterns?

While ID infants seem to show an initial delay, they more or less follow the developmental path of TD infants. Namely, after an initial delay in inter-subjective behavior they increase as do TD but a semester later. In the same way, ID children exhibit a significant increase of behaviors with objects during the first year, moving to catch up to the TD functioning. In contrast, AD children seem to develop otherwise. Especially, AD children show less orienting toward people in the first semester, and thereafter they exhibit a much smaller increase of seeking people behaviors than TD (whose score is multiplied by 4). As already described in a previous study [31], during the second semester there is an increase of orienting toward people and in receptive behaviors, especially smiling to people. But this increasing pattern, from an interactive point of view, appears to be *passive*, and after the first birthday these receptive behaviors dramatically decrease (to note that receptive behaviors remain stable both in TD and in ID children). Thus, it seems that the real marker for atypical social development is the weakness in initiating a social interaction: without the increase of social initiative the ability to be receptive and responding to others also becomes more scarce. Moreover, inter-subjective behaviors, even if globally lower, become specifically lower after the first birthday.

All these results are consistent with the hypothesis of a growing deviant development in AD [1] whereas children with ID show just a delay of social development, as illustrated in figure 7 summarizing the NMI values of non negative matrix factorization. This deviant development concerns also BB exploring object, which we did not find significant in the previous paper whose focus was on behaviors not on interaction context. Indeed, in the present study exploring object appears significantly reduced in the AD group as soon as the second half of the first year. This means that AD babies have less exploration of object inside the early interactive context, and that, unlike for TD (and ID), exploring object doesn't increase for AD after the first birthday. Thus the child does explore object but outside a real social interaction: we suggest that this pattern could be the expression of an early (and growing) lack of joint attention in AD. Joint attention is known to be deficient in older children with autism [32], and early lack of joint attention is correlated with a poor social interaction [33].

With regards to CG behaviors there are both differences and similarities as far as initiative and response. First of all, caregivers have toward their babies longer regulation up interaction and less gestural solicitation. We imagine that gestural solicitation becomes reduced because it fails to get a response; as a confirmation, in the previous paper [10] we described how CG soliciting by name decreases as a matter of the reduced orienting to name by AD babies. On the other hand, the high regulation up has a different meaning. First, CG Regulation up duration appears higher, in the first 6 months and in both pathological groups, only in the interactive context (it was not significant without the filter of interaction): that means that interactive moments are sustained both in AD and in ID by CG Regulation up; TD babies do not need a large amount of these CG behavior to express their sociality. Second, after the first birthday, regulation up remains significantly higher only for AD. We can hypothesize that while parents of both AD and ID feel from the first 6 months that their baby needs to be more stimulated, afterwards only parents of AD are confronted with a lack of social interest in their baby as he/she appears to enter into a clearer pathological process in the third semester. Indeed, AD children showed a lack of interest in people from the first 6 months, an increase of engagement (even if more passive) in the second semester, and then, after the first birthday, also a sharp decline of receptive meta-behaviors. Third, this special pattern of CG regulation up is associated, in the second semester, with the fact that parents go on touching their child to obtain a response (unlike TD children, there's no decrease of touching). The pattern composed of higher touching and longer regulation up still remains present in the second year when parents become more conscious of the difficulties to obtain a response.

In contrast, parental responses to inter-subjective behaviors do not differ from parents of TD babies. The few differences in quantity of CG responses in the first semester can be put down to lower babies' inter-subjective behaviors as far as a parental response needs a soliciting child. In sum, it seems that, except feeling that their baby needs to be stimulated, parents respond globally in the same way to their babies when he/she starts an interaction.

### Clinical implication for early detection of autism

Over the past 20 years much attention has been dedicated to behavioral indicators that will be present very early in life, certainly in infancy. Nevertheless, prospective (such as siblings studies) and retrospective (such has home videos studies) studies have not yet identified a clear prodrome that is a constellation of unfailing early warning signs indicating the development of a disease up to the time in which the clinical symptoms fulfill the required criteria for a diagnosis [3]. Our study adds some general lines useful to reach the objective of identifying prodrome of autism.

First, our interaction data base (i.e. extracting all sequences of caregiver behavior and infant behavior occurring within a time window of 3 seconds) has provided some significant findings which are detectable only during parent-infant interaction. Thus, we propose that the best way to study the emergence of autism should be based on interaction rather than on behaviors of each part of the dyad. Concepts such as synchrony [11], closely-fitting match [34] and mutual adaptation could provide a great deal of help to workers in the field of early detection of autism [35].

Second, our study shows a course of autism characterized by a decreasing atypical pattern in the second semester of life and afterwards an increasing loss of contact. This pattern, that we have named ‘fluctuating type of onset’ [36], does not seem unusual in non regressive autism as in our sample. This finding could be of seminal importance for both individualization of the right windows in screening programs (first six months of life or after the first birthday) and implementation of timely effective parent-infant training in a sensible period as the second semester of life does appear.

Third, we can confirm that much credence should be given to parents when they entrust their concerns to professionals (as shown by retrospective parental questionnaires [37], [38]). Moreover our research shows that parent listening can be implemented by some specific question and/or observation about the hyper-stimulating style of parent interaction toward their baby; in fact, we suggest that this particular attitude betrays the presence of an under-active baby (lack of initiative, inability to provoke or to anticipate other's aims, hypo-activity) which need to be stimulated. Thus through this pattern of interaction parents seem to feel very early that something is wrong in their baby - long before diagnosis. Although, even if the BB intergroup differences do not reach significance and then are not detectable for a stranger (i.e. the pediatrician), some dynamic changes like the significant longitudinal decrease of “receptive” meta-behavior after the first birthday should presumably be detectable for the child's relatives.

### Limits of this study

The first limitation is the sample size. As we used rigorous statistical methods taking into account the random subject effect and autocorrelation, we did not always obtain an analyzable, known distribution, and as scenes were very variable for a given infant (due to the great variability among scenes), some strong tendencies did not reach statistical significance; a larger sample would probably have allowed us more analyzable and/or significant results. Second, the analysis currently performed with our interactive filter highlighted the interactive dynamics without specifying the part played by each partner in the interaction. This would require additional analysis (e.g. response rate to a given stimulation) to determine this with accuracy and probably a larger sample. And last, only behavioral aspects of the stimulations were taken into account here, but qualitative emotional investment should be assessed as well, for example with the analysis of prosody (e.g., motherese); further research will focus on this question as we recently developed an algorithmic tool to assess motherese in home movies [27].

We conclude that using engineering methods to study social interaction in home movies has improved our understanding of early interactions. We can assume that, even if most BB behavior intergroup differences do not reach statistical significance and then are not detectable for a stranger [10], some interactive/dynamic changes should be detectable for the child's relatives. Here, the results suggest that deviant autistic behaviors appear before 18 months when studying interactive pattern. Furthermore, parents of AD and ID children feel (consciously or not) the lack of interactive initiative and responsiveness of their babies and try to increasingly supply soliciting behaviors. Thus we stress that credence should be given to parents' feeling as they recognize, long before diagnosis, the pathological process through the interactive pattern with their child. These findings could help early identification of AD by encouraging professionals to provide more attention to parents concerns and ways of coping with their child.
